# Footwear technology and biomechanical adaptations in ultramarathon running: a PRISMA-Guided narrative review integrating direct and laboratory evidence

**DOI:** 10.3389/fbioe.2025.1642555

**Published:** 2025-09-25

**Authors:** Zbigniew Waśkiewicz, Anna Akbaş, Tomasz Grzywacz, Zbigniew Borysiuk

**Affiliations:** ^1^ Institute of Sport Sciences, Jerzy Kukuczka Academy of Physical Education, Katowice, Poland; ^2^ Faculty of Physical Culture, Gdansk University of Physical Education and Sport, Gdańsk, Poland; ^3^ Faculty of Physical Education and Physiotherapy, Opole University of Technology, Opole, Poland

**Keywords:** trail running, ultramarathon, running economy, cushioning, foot strike pattern, fatigue, gaitadaptation

## Abstract

**Background:**

Footwear influences biomechanical strategy, fatigue response, and performance outcomes in trail and ultramarathon running. Yet, much of the current evidence remains fragmented across isolated laboratory trials and small-scale field studies.

**Objective:**

This review synthesizes findings from 20 verified studies and one preprint to examine how footwear properties—such as midsole cushioning, longitudinal bending stiffness (LBS), heel-to-toe drop, and shoe mass—influence running economy, gait mechanics, and fatigue-related adaptations in prolonged trail environments.

**Methods:**

A systematic synthesis was conducted across experimental, observational, and in-race studies involving trail or ultramarathon runners. Studies that assessed biomechanical, performance, or fatigue-related outcomes in the context of footwear design and terrain were included.

**Results:**

Footwear design was found to affect running economy and mechanical loading significantly, but the magnitude and direction of these effects were highly context-dependent. Stiffer shoes with advanced midsole geometry improved energy efficiency in trained runners under controlled conditions, while more compliant foams offered protective benefits during downhill and prolonged efforts. Foot strike patterns shifted dynamically in response to terrain slope and fatigue, with flatter landings and increased step frequency emerging as common compensatory strategies. Importantly, shoe materials degraded under racing conditions, altering their functional properties during the event.

**Conclusion:**

There is no universally optimal shoe for trail and ultramarathon running. Instead, performance and protection depend on how footwear features align with individual biomechanics, terrain demands, and the capacity for gait adaptation under fatigue. These findings support a move away from categorical shoe labels toward personalized, terrain-aware footwear strategies that evolve with the runner and the race. In practice, this means that athletes and coaches should prioritize adaptability across terrain and fatigue states rather than seeking a universally superior footwear model.

## 1 Introduction

Trail and ultramarathon running have seen an explosive rise in global participation over the past 2 decades. As competitive interest and event diversity have grown—from short alpine trail races to multiday ultramarathons—so too has attention on optimizing equipment to enhance performance and reduce injury risk. As the primary interface between runner and terrain, footwear modulates mechanical stress, energy expenditure, and gait adaptations during prolonged running across uneven surfaces (Jastifer and Hoffman, 2023; Kasmer et al., 2014). In this review, we distinguish between trail running (off-road running on variable terrain and elevation) and ultramarathons (any race longer than 42.2 km, regardless of surface). While many ultramarathons are run on trails, the two are not synonymous. Throughout this paper, we use the term ‘ultramarathon’ when referring specifically to distance, and ‘trail’ when emphasizing surface and elevation demands. Recent reviews also emphasize that footwear design can alter both biomechanics and injury risk, particularly when interacting with training load and surface (Sun et al., 2020)*.* Footwear terminology is clarified as follows: heel-to-toe drop refers to the difference in height between the heel and forefoot; stack height refers to the total cushioning thickness underfoot; and longitudinal bending stiffness describes the resistance to toe-off flexion.

Recent advances in running shoe technology have sparked renewed scientific and commercial interest in how design elements such as midsole cushioning, longitudinal bending stiffness (LBS), heel-to-toe drop, and shoe mass influence biomechanics and endurance performance. In contrast, road racing has driven much of this development, particularly the rise of so-called “super shoes.” This shift has reduced the relevance of the earlier minimalist–maximalist paradigm, as shoe performance is now driven more by integrated midsole–plate technologies than by cushioning extremes. Consequently, debates about minimalism versus maximalism have been replaced by evaluations of how super shoes alter biomechanics under endurance demands. Trail and ultramarathon running present unique demands. The cumulative mechanical load, frequent elevation changes, and variable terrain in long-distance trail events amplify the importance of footwear-related biomechanical efficiency (Giandolini et al., 2016b; Lloria-Varella et al., 2022). Equally important is the progressive neuromuscular fatigue that interacts with terrain and footwear. This third dimension not only modifies biomechanics but also determines how effectively athletes can adapt their gait strategies throughout ultra-endurance events.

Historically, the footwear-performance discourse has been framed around two competing paradigms: minimalist vs. maximalist shoes. Minimalist shoes, characterized by low cushioning and low heel-to-toe drop, were promoted for encouraging more “natural” foot strike patterns and reducing injury risk through improved proprioception and foot strength (Mo et al., 2021; Sinclair et al., 2015). Maximalist shoes, conversely, offer thick midsoles and are designed to attenuate impact forces—often used in long trail events to reduce fatigue and enhance comfort (Fritz et al., 2023; Shorten, 2024). More recently, “advanced footwear technology” (AFT), which incorporates carbon-fiber plates and energy-return foams, has introduced a third design philosophy centered on biomechanical efficiency and metabolic economy (Rodrigo-Carranza et al., 2024). This shift renders the earlier minimalist–maximalist dichotomy less central, as AFT integrates cushioning, stiffness, and energy return within a single system. Consequently, current debates focus on how foam–plate interactions modulate running economy and fatigue resistance in specific athletes and contexts, rather than on categorical shoe labels. Practically, this reframes footwear selection as a problem of matching integrated shoe mechanics to the runner’s characteristics and the demands of the terrain.

However, the evidence supporting these technologies is fragmented, particularly regarding application in trail and ultramarathon contexts. Studies on running economy and LBS are predominantly conducted on treadmills with elite or sub-elite athletes over short distances (Cigoja et al., 2022; Flores et al., 2019), raising concerns about ecological validity. Conversely, field-based studies on foot strike behavior, fatigue, and step kinematics in trail running often employ observational or case study designs with small sample sizes (Giandolini et al., 2016a; Kasmer et al., 2014). Furthermore, while minimalist shoes are often associated with midfoot or forefoot strike, trail-specific investigations reveal a predominant rearfoot strike pattern across most terrains and runners, even among users of minimalist footwear (Kasmer et al., 2014; Mo et al., 2021).

There is also increasing recognition that footwear effects may vary not only by shoe type but by interaction with terrain (uphill vs. downhill), runner characteristics (e.g., limb morphology, strength), and race duration (acute vs. cumulative fatigue effects) (Lloria-Varella et al., 2022; Rodrigo-Carranza et al., 2020). Shock attenuation strategies may shift under fatigue, influencing foot strike, step frequency, and soft tissue vibration dynamics (Giandolini et al., 2016b; Trama et al., 2023). However, the literature lacks a unified view that integrates lab-based mechanical findings with field-based adaptations in ultramarathon settings.

Despite the proliferation of research on footwear technology, no synthesis to date has systematically integrated experimental and real-world studies across the ultramarathon and trail running domains. Most prior reviews have focused on acute laboratory findings in road running populations, leaving a gap in understanding how footwear affects biomechanics, energy cost, and performance across varying terrains, fatigue states, and runner profiles.

This review aims to systematically synthesize empirical evidence on the biomechanical and performance effects of specific footwear characteristics, such as midsole cushioning, longitudinal bending stiffness (LBS), and advanced footwear technologies, in ultramarathon and trail runners. By integrating findings from in-race assessments and controlled laboratory studies, the review examines how these footwear variables interact with terrain conditions and fatigue to influence running economy, gait adaptations, and potential risk of injury.

## 2 Methods

This review adheres to the PRISMA (Preferred Reporting Items for Systematic Reviews and Meta-Analyses) guidelines, which comprise a 27-point checklist and a flow diagram (Haddaway et al., 2022). No preregistration was undertaken. Given that our review was entirely based on previously published articles, it did not necessitate ethical approval. This systematic review was not registered in any public review registry.

The research question guiding this review was structured using the PICO framework:

• Population (P): Adult participants engaged in ultramarathons and trail running, often under prolonged or fatigued conditions.
• Intervention (I): Various footwear features, including midsole cushioning, longitudinal bending stiffness (LBS), and advanced footwear technologies (AFT).
• Comparison (C): While not always explicitly defined across studies, comparisons typically involved different footwear models, terrain types (e.g., uphill vs. downhill), or states of fatigue versus non-fatigue.
• Outcomes (O): Primary outcomes included biomechanical adaptations (e.g., tibial shock, gait kinematics), running performance metrics (e.g., speed, oxygen cost), gait variability, and indicators of injury risk.

### 2.1 Search strategy

To identify and synthesize relevant peer-reviewed literature published between 2015 and 2024, a structured search strategy was employed to capture studies evaluating biomechanical and performance outcomes of footwear in ultramarathon and trail running contexts. To make the process transparent, we first searched PubMed, Scopus, and Web of Science using combinations of keywords for ‘ultramarathon’, ‘trail running’, and ‘footwear’. Although SciSpace was used as the initial aggregator platform, all potentially eligible records were cross-verified in PubMed and Scopus and supplemented by manual checks in Web of Science and reference lists, ensuring broad coverage and minimizing risk of omission. Two reviewers screened all abstracts, and full texts were checked against pre-specified inclusion and exclusion criteria. This ensured that both field-based ultramarathon studies and supportive laboratory work were captured systematically.

The search was conducted independently by two authors (Z.W. and A.A.) and focused on full-text, empirical studies verified for relevance and publication status. Grey literature, abstracts, and citation-only records were excluded to maintain scientific rigor. Studies were categorized as either direct evidence (ultramarathon or trail populations/field protocols) or supportive evidence (laboratory-based, road-running, or conceptual studies). Direct evidence informs external validity, whereas supportive evidence informs mechanistic understanding.

Search terms included combinations of keywords such as: “ultramarathon” AND “footwear”, “trail running” AND “shoe type”, “minimalist OR maximalist shoes” AND biomechanics, “cushioned shoes” AND “performance”, and “impact loading” AND “ultrarunners”. The literature search was performed using SciSpace (formerly Semantic Scholar), which aggregates peer-reviewed publications from sources such as PubMed Central, arXiv, and CrossRef. To enhance completeness, reference lists of included articles were manually screened to identify any additional relevant studies. Furthermore, all included articles were independently verified for full-text availability, publication status, and methodological relevance. The final database search was conducted in March 2025.

### 2.2 Selection criteria

To ensure both scientific quality and ecological relevance, studies were included based on the following criteria:

• Published in peer-reviewed journals between 2015 and 2024,
• Or available as full-text preprints that provided empirical data, fulfilled all inclusion criteria, and were independently verified for methodological adequacy,
• Available in full-text format, verified and archived,
• Explicitly focused on trail running, ultramarathon running, or biomechanical assessments in prolonged running conditions,
• Included empirical data on at least one of the following:
o Midsole cushioning, foam properties, or energy return
o Longitudinal bending stiffness (LBS)
o Foot strike pattern or distribution
o Tibial shock or soft tissue vibrations
o Step frequency, kinematics, or gait adaptations under fatigue
o Terrain-specific performance (e.g., downhill or uphill)
o Advanced footwear technology (AFT)

Studies were excluded if they:

1. Focused solely on recreational road running without a trail-specific application.
2. Used treadmill-only protocols without a biomechanical context applicable to trail settings.
3. They were purely theoretical or commentary articles without experimental or field data.

**FIGURE 1:**
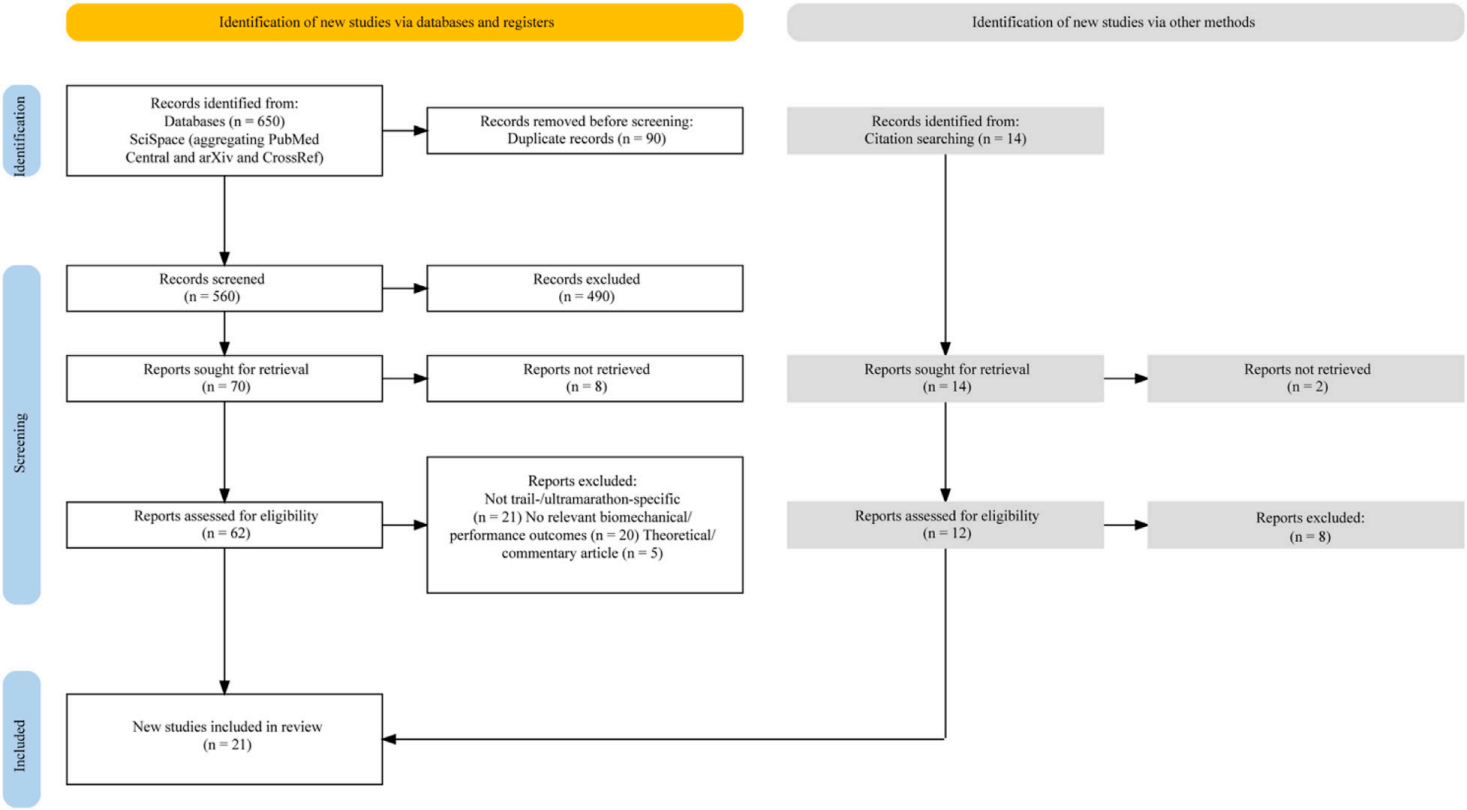
PRISMA 2020 flow diagram for study selection.

A single preprint (Matties et al., 2024) was included based on its empirical focus, methodological completeness, and relevance to the review question. It was included due to its unique longitudinal design. To mitigate bias, it underwent the same NOS-based quality appraisal as published studies and was explicitly identified as a preprint in the Results and Discussion section. A PRISMA flow diagram (Figure 1) illustrates the identification, screening, eligibility, and inclusion of studies, with reasons for exclusion at each stage (Haddaway et al., 2022).

### 2.3 Synthesis approach

Given the methodological and clinical heterogeneity across included studies, in terms of design, participant characteristics, interventions, and measured outcomes, a meta-analysis was not feasible. Instead, a structured thematic synthesis was conducted to organize and interpret the findings across the included studies. Studies were categorized based on primary variables of interest, which enabled consistent cross-study comparison within defined domains. These domains included:

• Footwear properties (e.g., cushioning, LBS, foam fatigue)
• Performance and economy (e.g., oxygen cost, propulsion force, running speed)
• Kinematics and ground impact (e.g., tibial shock, step frequency)
• Terrain-specific adaptations (e.g., uphill/downhill gait, strike variability)
• Fatigue and post-race compensation (e.g., muscle soreness, soft tissue vibration)

Each included study was assessed for methodological quality using design-appropriate tools. Observational studies were evaluated using the Modified Newcastle-Ottawa Scale (NOS). In contrast, experimental and case studies were evaluated for the clarity of methods, validity of outcome measurement, and reporting of statistical results (Wells et al., 2001). Risk of bias and limitations were documented in the Results and Discussion sections.

## 3 Results

The flowchart summarizes the study identification, screening, and inclusion process for this systematic review. A total of 650 records were initially identified through database searches (SciSpace, aggregating PubMed Central, arXiv, and CrossRef), with 90 records removed via automated ineligibility filters. After screening 560 titles and abstracts, 70 full-text reports were retrieved, of which eight could not be obtained. Of the 62 reports assessed for eligibility, 46 were excluded because they did not meet the inclusion criteria. An additional 14 records were identified through citation searching, resulting in 12 full-text assessments and two exclusions. In total, 21 studies were included in the final qualitative synthesis. Of the 21 included studies, 10 were classified as direct evidence and 11 as supportive evidence (see Tables 1, 2; Figure 1).

**TABLE 1:** Characteristics of included studies classified as direct (ultramarathon/trail-specific) or indirect (laboratory or road-based) evidence.

(Author, year)	Population/setting	Design & terrain	Key outcomes
Direct evidence – ultramarathon/trail field studies (n = 10)			
Kasmer et al. (2014)	161-km ultramarathon runners	Field observational	Foot strike patterns, performance
Giandolini et al. (2016a)	World-class trail athletes	Field race analysis	Step frequency ↑, impact attenuation, stability
Giandolini et al. (2016b)	110-km mountain ultramarathon	Pre–post-race	Ankle ROM ↓, strike shifts, fatigue
Defer et al. (2019)	Trail race (downhill trials)	Field, downhill focus	Drop effects, descent performance
Lloria-Varella et al. (2022)	38-km trail race	Pre–post footwear test	Midsole degradation, tibial shock
Mo et al. (2021)	Trail runners	Uphill/downhill protocols	Strike pattern changes with slope
Mo et al. (2021), field)	High-altitude ultramarathon	Field and lab	Minimalist vs. maximalist, no strike diff
Trama et al. (2023)	40–171-km ultramarathons	Pre–post muscle vibration	Vastus lateralis fatigue, STV ↓
Rodrigo-Carranza et al. (2023)	National trail runners	Field trials	LBS effects by fitness level
Rodrigo-Carranza et al. (2024)	Trail endurance	Experimental	LBS ↑ → energy cost ↓
Indirect evidence – laboratory/road-runner studies (n = 10)			
Cigoja et al. (2022)	Trained road runners	Lab treadmill	Joint work redistribution, stiffness
Flores et al. (2019)	Recreational runners	Lab	Neuromuscular activation, energy return
Martinez et al. (2024)	Competitive female runners	Lab “super shoe” test	Running economy ↑, joint mechanics
Fritz et al. (2023)	Elite ultra-trail (single case)	Lab, treadmill	Midsole stiffness ↓ → kleg ↑, economy ↑
Shorten (2024)	Theoretical/experimental	Conceptual review	Critique of “energy return” paradigm
Rodrigo-Carranza et al. (2020)	Road runners	Lab	Shoe mass ↑ → oxygen cost ↑
Mo et al. (2021), lab arm)	Runners	Controlled	Minimalist vs. maximalist biomechanics
Zhang & Vanwanseele (2017)	Habitual footwear users	Ultrasound study	Foot soft-tissue morphology
Sinclair et al. (2015)	Mixed population	Lab/road crossover	Biomechanical responses
Jastifer and Hoffman (2023)	Mixed population	Lab/road crossover	Complementary performance findings
Other – preprint (n = 1)			
Matties et al. (2024), preprint)	Advanced runners	Training study, lab	Midsole/energetic responses; not peer-reviewed

**TABLE 2:** Evidence on footwear effects in ultramarathon and trail running direct vs. indirect sources.

Direct evidence – ultramarathon/trail field studies (n = 10)	Main findings	Indirect evidence – laboratory or road-running studies (n = 11)	Main findings
Giandolini et al. (2016a)	World-class trail runners adapted stride frequency and reduced impact peaks under fatigue	Fritz et al. (2023)	Elite ultra-trail runner: softer midsole improved economy on level and downhill
Giandolini et al. (2016b)	110-km ultra: reduced ankle ROM, flatter strike patterns post-race; fatigue-driven adaptations	Flores et al. (2019)	High energy-return midsoles changed activation (↓ biceps femoris pre-activation, ↑ economy)
Kasmer et al. (2014)	161-km ultra: 85% rearfoot strike; strike variability predicted top finishers	Martinez et al. (2024)	Female competitive runners: super shoes improved joint mechanics and economy
Defer et al. (2019)	Low-drop shoes improved downhill speed and promoted forefoot strike	Shorten (2024)	Energy return is systemic (timing/geometry), not spring-like recovery
Lloria-Varella et al. (2022)	38-km trail: midsole stiffness ↑, cushioning ↓; tibial shock patterns altered	Rodrigo-Carranza et al. (2023)	High LBS benefited elites at faster speeds; less benefit in sub-elite runners
Trama et al. (2023)	40–171 km ultras: reduced thigh vibration damping, neuromuscular fatigue markers	Rodrigo-Carranza et al. (2024)	LBS reduced energy cost, delayed ankle→knee redistribution
Mo et al. (2021), field)	Minimalist vs. maximalist: no difference in strike or tibial acceleration on trail slopes	Cigoja et al. (2022)	Stiffer shoes preserved ankle-dominant propulsion, delaying fatigue effects
Mo et al. (2021), field)	Uphill increased forefoot strike regardless of shoe; terrain dictated adaptations	Mo et al. (2021), lab)	No biomechanical differences minimalist vs. maximalist in rearfoot strikers
Rodrigo-Carranza et al. (2020)	Shoe mass +100 g ↑ O_2_ cost in race-relevant conditions	Mo et al. (2021), lab)	Uphill vs. downhill: strike shifts primarily terrain-driven
Zhang & Vanwanseele (2017)	Long-term footwear use altered intrinsic foot morphology (ultrasound evidence)	Preprint: Matties et al. (2024)	Shoe–terrain interactions in simulated ultra

### 3.1 Quality assessment and risk of bias

All 21 studies included in this review were evaluated for methodological quality using a modified version of the Newcastle-Ottawa Scale (NOS), adapted for biomechanical and performance-based research. The NOS consists of three domain: Selection (maximum 4 points), Comparability (maximum 2 points), and Outcome/Exposure Assessment (maximum 3 points), resulting in a total score ranging from 0 to 9. In this review, the NOS was modified to emphasize biomechanical criteria such as sample characterization, instrumentation validity, and reproducibility of outcome measures, following precedents in prior sports biomechanics reviews. Studies with lower scores were not excluded; however, their findings were weighed cautiously in the synthesis, and limitations were explicitly noted in the discussion.

**FIGURE 2:**
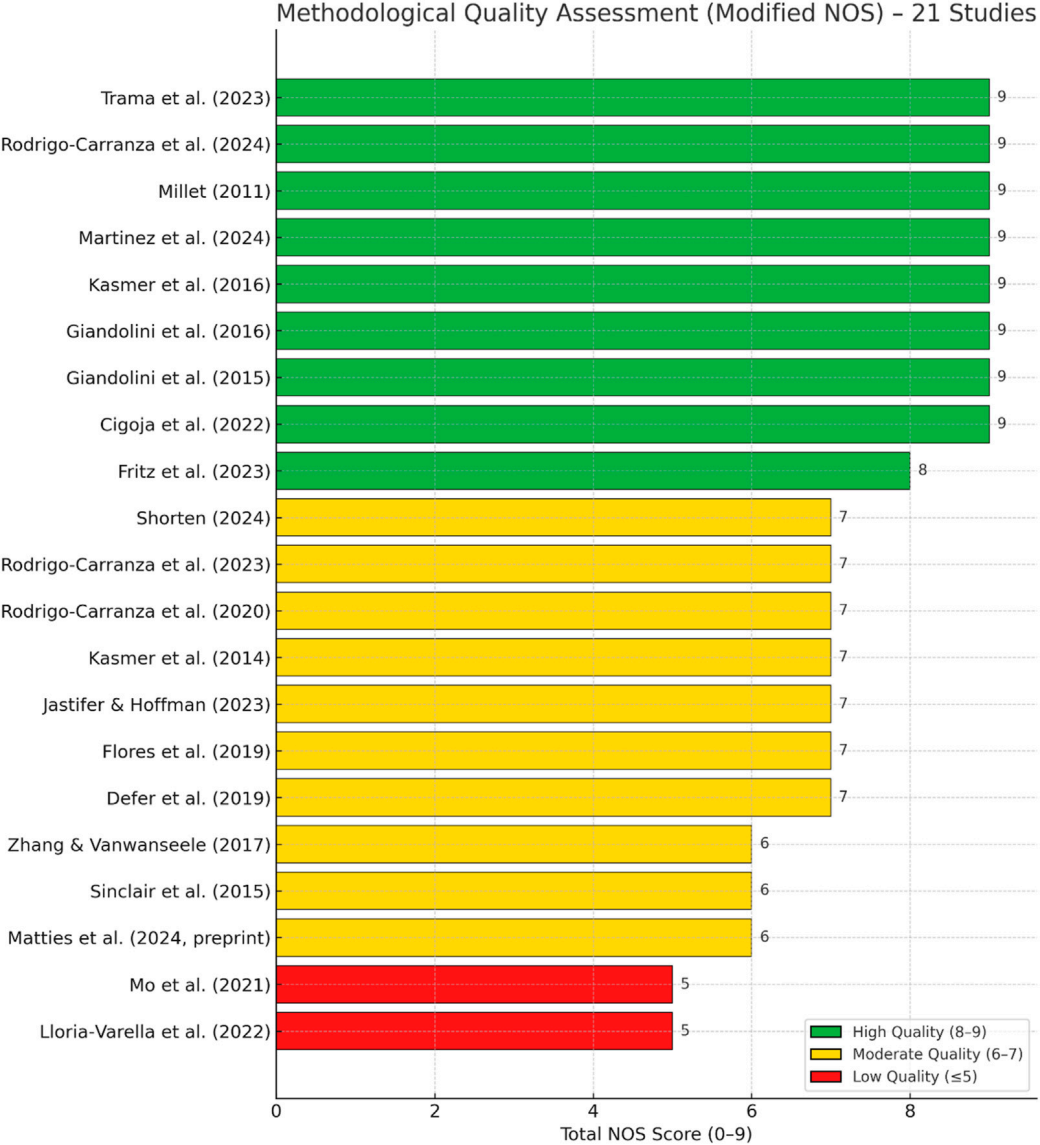
Quality assessment of included studies using the Modified Newcastle-Ottawa Scale (NOS).

Based on their total scores, studies were classified into three quality tiers: high quality (8–9 points), moderate quality (6–7 points), and low quality (≤5 points). As shown in Figure 2, total scores ranged from 5 to 9 across the included articles. Detailed domain-level scores and classifications are presented in Supplementary Table S1. Of the 20 included studies, eight were rated as high quality, 10 as moderate quality, and two as low quality. High-quality studies—such as Cigoja et al. (2022), Flores et al. (2019), Giandolini et al. (2016b), Kasmer et al. (2014), Martinez et al. (2024), Rodrigo-Carranza et al. (2023), Rodrigo-Carranza et al. (2024), and Trama et al. (2023)—were characterized by well-defined populations, intra-individual study designs, and the use of validated biomechanical and physiological outcome measures. Moderate-quality studies typically lacked randomization, featured smaller sample sizes, or provided only limited outcome data. Two studies—Mo et al. (2021) and Lloria-Varella et al. (2022)—were rated as having low quality due to the limited reporting of participant characteristics, the absence of comparator conditions, and methodological ambiguity in the outcome analysis.

Despite some variability, the overall evidence base demonstrated acceptable methodological rigor. Most studies performed well in the Selection domain. In contrast, variability in the Comparability and Outcome/Exposure domains was primarily driven by study design (e.g., case reports, non-randomized trials, observational cohorts). These findings support the reliability of the included literature and its suitability for structured narrative synthesis.

### 3.2 Footwear mechanical properties

Footwear design elements shape performance, biomechanics, and fatigue management in trail and ultramarathon running. Unlike road environments, trail terrains introduce fluctuating mechanical demands due to elevation changes, surface irregularity, and prolonged impact exposure. Consequently, the interaction between midsole composition, structural stiffness, shoe geometry, and the runner’s biomechanical strategy becomes essential to understanding performance outcomes. These observations are consistent with systematic evidence indicating that footwear design has a significant influence on running economy and performance outcomes across distance running populations (Fuller et al., 2015). This section synthesizes findings across three core footwear features: midsole cushioning and energy return, longitudinal bending stiffness (LBS), and shoe geometry (mass, heel-to-toe drop, and stack height).

#### 3.2.1 Midsole cushioning and energy return

Midsole cushioning serves dual roles in shock attenuation and modulating energy efficiency. Recent evidence suggests that more compliant midsoles may provide a metabolic advantage under endurance conditions by altering muscle activation and enhancing energy management. In a controlled case study, Fritz et al. (2023) demonstrated that decreasing midsole stiffness resulted in increased leg stiffness (kleg) and improved running economy during both level and downhill running in an elite ultra-trail runner. This relationship is crucial in trail contexts, where mechanical efficiency and muscular protection must be maintained over extended periods. While this case study provides valuable insights, it cannot be generalized to all runners. Responses to midsole compliance appear highly individual, with some athletes showing improved economy while others may not benefit or may even experience maladaptive muscle activation strategies. Broader samples are therefore needed to confirm whether these effects extend beyond elite individuals.

Complementing this, Flores et al. (2019) found that runners benefiting from high energy return midsoles exhibited distinct neuromuscular strategies, including lower biceps femoris pre-activation and more economical activation during the braking and push-off phases. These adaptations suggest that some runners may leverage midsole mechanical properties to redistribute workload and optimize energy use. Martinez et al. (2024) similarly observed that super shoes enhanced running economy and joint mechanics in competitive female runners, highlighting the relevance of combined foam and plate technologies in performance footwear.

Despite these benefits, the notion of “energy return” in footwear remains conceptually debated. In a critical examination, Shorten (2024) argued that midsoles are not “spring-like” devices in the mechanical sense. Instead, energy return is a system-level phenomenon influenced by timing, geometry, and force transmission, with actual elastic recovery contributing less than is often assumed. His findings highlight that improvements in running economy are not the result of literal energy recycling but rather stem from optimized leverage and force direction during push-off.

Moreover, Lloria-Varella et al. (2022) demonstrated that cushioning performance deteriorates over long trail runs. After a 38-km trail race, midsole materials exhibited increased stiffness and reduced thickness, altering tibial shock transmission even without visible changes in running mechanics. This finding highlights the importance of considering midsole fatigue and degradation during ultra-distance events, particularly in relation to protective function and injury risk.

Although these findings emphasize cushioning as a key factor in energy efficiency, most evidence derives from single-athlete case studies or mixed road–trail protocols. The ecological validity for mass ultramarathon participation remains limited, and inter-individual responses appear highly variable. Future trials should incorporate in-race monitoring of shoe degradation to link laboratory observations with real-world fatigue adaptations.

#### 3.2.2 Longitudinal bending stiffness (LBS)

Research on advanced racing shoes indicates that carbon-plate integration and compliant foams can reduce energetic cost in distance running, a principle also relevant to trail contexts (Hoogkamer et al., 2018). Integrating carbon plates and other stiffening agents into shoe midsoles has drawn attention for their potential to enhance running economy through increased longitudinal bending stiffness. While initially studied in road contexts, trail-specific evidence supports their application in endurance trail running.

In a pivotal study, Rodrigo-Carranza et al. (2024) found that increasing LBS reduced energy cost during running, with the most significant benefits observed in trained runners at race-relevant speeds. However, these advantages are not universal. Recreational runners may experience limited benefits, and in some cases, stiffer shoes can disrupt coordination or increase distal fatigue. This highlights that the effectiveness of LBS is strongly influenced by the athlete’s experience and neuromuscular profile.

This aligns with the work of Cigoja et al. (2022), who reported that stiffer footwear delayed the onset of joint work redistribution from the ankle to the knee, a shift commonly associated with fatigue. Maintaining ankle-dominant propulsion for longer may reduce the recruitment of proximal musculature and preserve neuromuscular function across prolonged efforts. Notably, Rodrigo-Carranza et al. (2023) also highlighted that the effects of LBS are not universal. Benefits were greater in elite athletes and at faster speeds, suggesting that responsiveness to stiffness is partly moderated by fitness level and gait economy. Similarly, Flores et al. (2019) emphasized that individuals with greater leg stiffness and a proximal muscle activation strategy (e.g., greater vastus medialis use) may respond more positively to LBS-enhanced shoes. These findings suggest that LBS may enhance running efficiency, particularly in trained runners who exhibit neuromechanical patterns compatible with stiffer midsoles. However, mismatched stiffness can disrupt coordination, increase distal fatigue, or impair proprioception on technical terrain.

While promising, these findings expose essential limitations. First, most trials involve small, homogeneous groups of well-trained runners, making it challenging to extrapolate the results to recreational ultramarathoners. Second, none of the studies systematically examined terrain variability, meaning that coordination or proprioceptive deficits induced by stiffness may be underestimated in technical races. Consequently, recommendations on LBS remain conditional and population-specific.

#### 3.2.3 Shoe mass, heel-to-toe drop, and stack height

While often overshadowed by high-tech features, traditional footwear variables like shoe mass, drop, and stack height remain foundational to performance outcomes, especially in ultramarathon settings. Rodrigo-Carranza et al. (2020) confirmed that adding just 100 g per shoe significantly increased oxygen cost, reinforcing longstanding data on shoe mass and running economy. This relationship is particularly salient in trail races, where additional shoe weight may be justified for traction or protection but accumulates a metabolic penalty over long distances. For instance, Perl et al. (2012) showed that habitual minimalist runners exhibited improved running economy compared with when running in conventional shoes, underscoring the role of long-term footwear exposure. Running economy is determined by multiple interacting footwear and biomechanical factors, including shoe mass and geometry, which have been systematically reviewed in the broader literature (Barnes and Kilding, 2015).

Heel-to-toe drop has a pronounced influence on gait mechanics and terrain-specific performance. In a downhill performance trial, Defer et al. (2019) found that runners wearing low-drop shoes adopted more forefoot strikes and completed descents more quickly than those wearing higher-drop shoes. These findings support the idea that lower drop facilitates more dynamic, anterior landings, which are especially useful in steep, technical terrain. Conversely, higher drop shoes may encourage rearfoot striking and potentially reduce loading on the Achilles tendon. This tradeoff must be managed in consideration of the runner’s characteristics and the course profile.

Stack height contributes to both energy absorption and proprioceptive input. High-stack shoes may offer superior cushioning and reduced impact peaks, particularly relevant in ultra-distance events, yet at the cost of lateral stability. These variables cannot be optimized simultaneously: maximizing cushioning often compromises ground feel and stability, whereas prioritizing stability may reduce comfort and impact attenuation. Coaches and athletes should therefore treat drop and stack height as trade-offs that must be balanced against the demands of the terrain and individual tolerance.

This may increase the risk of falls or impair ground feel in technical sections. While Mo et al. (2021) did not find significant biomechanical differences between minimalist and maximalist shoes in rearfoot strikers, their data suggest that stack height effects are terrain- and runner-dependent rather than universally prescriptive.

Overall, shoe mass, drop, and stack height represent accessible levers for modifying performance, yet most evidence derives from controlled slopes or laboratory settings. Their interaction with cumulative fatigue and highly variable ultramarathon terrain remains poorly studied. In practice, this means that recommendations must be contextually relevant, striking a balance between energy costs, stability, and injury risk.

#### 3.2.4 Kinematics and foot strike adaptations

The foot strike pattern—defined by the portion of the foot that contacts the ground first during the initial stance—is a critical variable in running biomechanics. It influences joint loading pathways, muscle recruitment, shock attenuation strategies, and energy distribution throughout the gait cycle. While early studies linked non-rearfoot strike (non-RFS) patterns, such as midfoot and forefoot strikes, to reduced impact peaks and improved running economy, trail and ultramarathon settings present more complex and variable conditions where foot strike behavior often defies conventional assumptions.

Contrary to expectations that minimalist or low-drop footwear universally promotes a midfoot or forefoot strike, field studies suggest that rearfoot striking remains the dominant pattern in trail ultramarathons across various shoe and terrain types. For instance, in a 50-km trail race, Kasmer et al. (2014) observed that 85.1% of participants used a rearfoot strike, even among runners wearing minimalist shoes (Table 3). Similarly, in a high-altitude ultramarathon, Mo et al. (2021) reported no significant difference in foot strike pattern or peak tibial acceleration between minimalist and maximalist shoe users during uphill, level, or downhill running. Critical perspectives on barefoot and minimalist running highlight that the strike pattern alone does not determine injury risk or efficiency, but interacts with footwear and terrain demands (Nigg and Enders, 2013). One likely explanation is the unstable and unpredictable nature of trail terrain, which encourages runners to maximize stability and surface contact. Rearfoot striking may also reflect instinctive protective strategies during descents and states of fatigue, where runners prioritize safety over efficiency.

**TABLE 3:** Foot strike pattern, terrain, and shoe type.

Study	Terrain type	Shoe type/feature	Observed strike pattern	Performance link
Mo et al. (2021)	Uphill, downhill, level	Minimalist vs. Maximalist	Predominantly RFS; uphill induced more MFS/FFS	No clear performance difference by shoe or strike
Kasmer et al. (2016)	Trail (50-km race)	Minimalist vs. traditional	RFS in 85%; minimalist users had slightly less RFS	Faster runners wore minimalist shoes more often
Kasmer et al. (2014)	Trail (161-km race)	Not specified	RFS most common; increased variability in top finishers	Strike variability (not type) associated with better performance
Giandolini et al. (2016b)	Trail (110-km race)	Trail-specific racing shoes	Shift from FFS/MFS to flatter/RFS under fatigue	Considered adaptive; protective of fatigued muscles
Defer et al. (2019)	Downhill	Low drop vs. standard drop	Lower drop → ↑ FFS use	↓ time with low drop due to more anterior landing
Giandolini et al. (2016a)	Trail (45-km race)	Trail racing shoes	High intra-run variability in elite athlete	Suggested flexibility/adaptability contributes to success
Martinez et al. (2024)	Treadmill	Super shoes vs. traditional	Foot strike shifted with shoe type	Linked to improved RE via joint mechanics

Terrain slope strongly modulates foot strike behavior. Uphill running tends to encourage a shift toward midfoot or forefoot strike, as runners naturally adopt shorter, more vertical strides for mechanical leverage. Mo et al. (2021) and Defer et al. (2019) found significant increases in forefoot strike percentages during uphill segments, regardless of shoe type. Conversely, downhill running promotes rearfoot striking, likely as a protective adaptation to manage braking forces and control descent speed, mainly when muscle fatigue accumulates (Giandolini et al., 2016a).

Interestingly, studies suggest that foot strike may not be a strong independent predictor of performance in ultramarathon events. Kasmer et al. (2014) observed no direct relationship between strike type and finishing times in a 161-km race. However, they noted that top finishers tended to exhibit greater variability in foot strike patterns, suggesting that adaptable movement strategies—rather than fixed biomechanical profiles—may be more beneficial in endurance trail events. Moreover, foot strike may serve as an indicator of cumulative fatigue or neuromuscular compensation. In a biomechanical study of a 110-km mountain ultramarathon, Giandolini et al. (2016b) found that runners generally adopted flatter foot landings after prolonged exertion, regardless of their pre-race strike type. This adaptation was interpreted to reduce localized loading on fatigued structures such as the ankle plantar flexors.

Taken together, these findings highlight adaptability as a key determinant of success. Yet, most available studies remain descriptive and lack direct shoe comparisons under fatigue. Without controlled interventions, it is difficult to disentangle whether strike variability is driven by footwear, terrain, or fatigue alone. This limits the strength of causal inferences. From a practical perspective, this reinforces the importance of training athletes to adjust their strike strategies according to terrain and fatigue levels. Rather than prescribing a fixed strike pattern, coaches may encourage athletes to practice switching between landing strategies in varied trail conditions, thereby enhancing resilience and performance under ultramarathon demands.

### 3.3 Fatigue and shoe degradation

Prolonged trail and ultramarathon running impose substantial cumulative stress on the musculoskeletal system (Table 4). As race duration increases, fatigue-related neuromuscular impairments emerge, triggering compensatory adjustments in gait mechanics, impact attenuation strategies, and tissue loading profiles. Understanding how fatigue interacts with footwear design and terrain is critical for interpreting performance outcomes and mitigating injury risk in ultramarathon environments. In this review, fatigue is primarily referred to as cumulative neuromuscular fatigue during prolonged running. When central, peripheral, or acute fatigue are discussed, these are explicitly specified. This dual degradation—of both muscle and midsole—may interact: as cushioning stiffens, fatigued musculature is forced to absorb greater impact, potentially increasing instability and injury risk late in races. While causal trials are lacking, studies suggest a cumulative link between material fatigue, biomechanical adaptation, and musculoskeletal load.

**TABLE 4:** Fatigue-induced biomechanical and material adaptations.

Study	Race distance/load	Fatigue measure	Footwear context	Key adaptations observed
Giandolini et al. (2016b)	110 km	Pre/post neuromechanical testing	Trail racing shoes	↓ ankle ROM, ↑ step frequency, flatter strike in fatigued state
Trama et al. (2022)	40–171 km	VL/GAS soft tissue vibration (STV)	Consistent race shoes	↓ VL vibration frequency/damping post-race, no ↑ in impact force
Lloria-Varella et al. (2022)	38 km	Shoe mechanical testing, tibial acceleration	Personal vs. control shoes	↑ stiffness, ↓ cushioning thickness, altered ML tibial acceleration
Giandolini et al. (2016a)	45 km	Continuous gait observation	Trail racing shoes	↑ step frequency, smoother landing as fatigue progressed
Kasmer et al. (2014)	161 km	Gait variability (strike pattern changes)	Mixed shoes	↑ variability in strike pattern with duration (top performers)
Matties et al. (2024)	8-week training intervention	Post-training biomechanical re-testing	AFT footwear	Improved kinematics and shock management after AFT exposure

#### 3.3.1 Neuromuscular fatigue and kinematic compensation

In a comprehensive study of a 110-km mountain ultramarathon, Giandolini et al. (2016b) reported significant reductions in ankle range of motion and an increase in step frequency post-race. These adaptations likely represent attempts to minimize the eccentric loading of fatigued muscles, particularly the plantar flexors and knee extensors. Notably, runners who began the race with a non-rearfoot strike pattern shifted toward a flatter or even rearfoot landing at the end, suggesting that fatigue may override initial foot strike preferences in favor of more conservative mechanics.

Complementing these results, Trama et al. (2023) investigated runners before and after 40- to 171-km races, assessing changes in soft tissue vibration (STV) and foot-ground impact. While gastrocnemius medialis vibration patterns remained stable, vibration amplitude and damping in the vastus lateralis decreased post-race, indicating muscular fatigue and possibly impaired shock-absorbing capacity in the thigh. Interestingly, these biomechanical changes occurred without a corresponding increase in measured ground impact forces, suggesting that runners maintained or adjusted their gait to avoid elevating tibial loads despite systemic fatigue. These adaptations are consistent with theoretical models of fatigue in ultramarathons, where neuromuscular decline shapes pacing and biomechanical strategies (Millet, 2011).

These results indicate that footwear can buffer, but not prevent, fatigue-driven biomechanical changes. Still, nearly all evidence comes from observational studies of race without randomization or control conditions, which prevents the causal attribution of protective shoe effects. For coaches, these findings imply that monitoring quadriceps fatigue, for instance, through wearable vibration or EMG sensors, could help anticipate when compensatory gait changes are likely to emerge. Such monitoring may support individualized pacing or recovery strategies in long trail events.

#### 3.3.2 Shoe fatigue and material degradation

In addition to biological fatigue, midsole materials undergo degradation during prolonged races. In a 38-km trail race study, Lloria-Varella et al. (2022) found that personal footwear exhibited increased stiffness, reduced cushioning thickness, and decreased energy dissipation after the event. Shifts in mediolateral tibial acceleration patterns accompanied these changes, although participants’ stride frequency and contact times remained stable. This decoupling between ground impact metrics and foot-ground kinematics implies that midsole aging affects internal load transfer even when external mechanics appear unchanged.

Additionally, habitual exposure to specific footwear types may result in structural adaptations of the foot’s soft tissues, as demonstrated by Zhang and Vanwanseele (2017) in their ultrasound-based morphological study. The interplay between muscle and midsole fatigue is particularly relevant in multi-hour events where the cumulative degradation of biological and mechanical shock absorption systems may elevate injury risk or impair performance.

This dual fatigue—encompassing both muscle and material—remains underexplored, as no studies have directly modeled its combined effects. Current evidence only indirectly suggests that as midsoles stiffen, fatigued musculature is forced to absorb greater loads, a mechanism that could predispose runners to late-race injuries. From a practical perspective, this dual fatigue highlights that athletes should expect their footwear to change functionally during long races. Coaches may integrate this awareness into training by exposing runners to prolonged sessions in partially worn shoes, thereby simulating the coupled effects of muscle and material fatigue.

#### 3.3.3 Adaptive stability strategies

Fatigue-induced gait changes also appear to serve a stabilizing function in uncertain or degraded terrain. Giandolini et al. (2016a) observed that world-class trail athletes displayed increased step frequency and smoother, lower-impact strides over time, interpreted as a strategy to reduce the mechanical cost of oscillatory movement and improve balance. Similarly, Kasmer et al. (2014) found that top ultramarathon finishers exhibited greater variability in strike pattern late in the race, suggesting that movement flexibility may be an adaptive mechanism for preserving efficiency and stability under fatigue.

Collectively, these adaptations underscore that fatigue should be viewed not only as a decline in performance but also as a trigger for compensatory strategies. However, almost no research has linked these strategies to shoe-specific effects, meaning that our understanding of footwear’s role in adaptive stability remains incomplete.

### 3.4 Performance outcomes

The combined evidence on footwear properties, kinematics, and fatigue ultimately feeds into performance outcomes. Yet, performance in ultramarathons is emergent, influenced by the interaction of shoe mechanics, terrain, pacing, environmental stressors, and individual adaptation. Current studies report mixed findings: while some demonstrate metabolic savings or delayed fatigue with specific shoe features, others find no consistent performance advantage once race variability is considered.

Thus, although footwear technology influences biomechanics and fatigue, performance outcomes remain speculative. The heterogeneity of study designs, populations, and race contexts precludes firm conclusions. At present, the most reliable applied principle is that no single shoe configuration optimizes performance for all runners or terrains; adaptability and individual matching are key.

## 4 Discussion

This review highlights the complex and context-dependent role of footwear in shaping running biomechanics, energy efficiency, and fatigue response in trail and ultramarathon environments. Rather than identifying a single ideal shoe configuration, the evidence points to a spectrum of interactions between footwear properties, individual biomechanics, and environmental conditions. These interactions are not static; they evolve throughout an event and respond to the cumulative demands placed on both the runner and the shoe.

One of the most notable patterns emerging from the literature is the adaptability of gait mechanics in response to fatigue and varying terrain conditions. Runners commonly exhibit shifts in foot strike pattern, cadence, and joint kinematics throughout a race. For example, flatter foot landings and increased step frequency have been observed late in ultramarathon efforts, regardless of a runner’s initial strike preference. These changes are interpreted as compensatory adjustments designed to manage muscular fatigue and maintain postural control. Notably, such adaptations appear to be influenced by terrain gradient as well, with uphill segments favoring midfoot or forefoot strike and downhill running eliciting more rearfoot-dominant strategies.

Footwear design can influence how effectively these adaptations occur. Features such as longitudinal bending stiffness and midsole cushioning have demonstrated the potential to reduce energy cost and delay muscular fatigue. However, their benefits are highly dependent on context. While some runners may experience improvements in running economy with stiffer or more responsive midsoles on flat or predictable terrain, the same features may become less advantageous—or even detrimental—on unstable or highly technical trails. This variability suggests that the effectiveness of footwear design is not universal, but conditional on the interaction between shoe mechanics and the biomechanical demands of the terrain. Equally important, runners must develop sensorimotor strategies to compensate for shoe-induced changes actively. Field studies have shown that when footwear alters ground feel or ankle leverage, athletes rely more heavily on rapid proprioceptive feedback to maintain their balance. This highlights that stability in trail ultramarathons is co-produced by both shoe mechanics and the nervous system, rather than solely by footwear properties.

In addition to influencing biomechanics, footwear is itself subject to change over time. Several studies indicate that shoe materials degrade throughout a race, resulting in reductions in cushioning performance and alterations in stiffness that affect the shoe’s ability to attenuate impact. These changes may further interact with the runner’s physiological fatigue, compounding the challenge of maintaining efficient and safe movement patterns. Despite this, many runners and practitioners continue to evaluate footwear based solely on pre-race characteristics, overlooking the possibility that performance may change significantly during the race due to material fatigue.

Given these findings, categorical recommendations—such as choosing minimalist versus maximalist shoes—appear overly simplistic. The review instead supports a more individualized and context-aware approach to footwear selection. Factors such as runner experience, gait variability, terrain profile, race duration, and prior training exposure should all inform footwear decisions. Moreover, training programs may benefit from integrating terrain-specific gait adaptation strategies and familiarization with race-day footwear to enhance performance and reduce the risk of injury.

Taken together, the evidence supports a broader view of footwear not as a fixed solution, but as a tool that must align with both the athlete’s capabilities and the environmental challenges of long-distance trail running. The most effective footwear may be that which enables runners to adjust safely and efficiently across conditions, rather than enforce a specific biomechanical pattern throughout the race.

## 5 Practical implications and recommendations

Practical applications must consider how footwear interacts with biomechanics, fatigue, and terrain in ultramarathon running. While certain shoe features—such as increased longitudinal bending stiffness or compliant midsoles—have shown promise in improving running economy or reducing muscular fatigue, their benefits are not universally applicable. Instead, the evidence suggests optimal performance outcomes hinge on matching footwear characteristics to individual neuromechanical profiles, terrain demands, and race durations (Table 5).

**TABLE 5:** Practical implications and terrain-specific recommendations.

Footwear feature	Biomechanical effect	Best applied use	Caution/limitation
Low Cushioning (Minimalist)	↑ Proprioception, ↑ tendon loading, ↓ weight	Technical terrain, short races, trained runners	↑ Achilles load; not protective over long durations (Sinclair et al., 2015)
High Cushioning (Maximalist)	↓ Impact peaks, ↑ comfort, ↓ proprioception	Ultra races, rocky descents, fatigue-prone runners	May impair stability on uneven terrain (Mo et al., 2021)
Low Heel-to-Toe Drop	Promotes anterior strike, ↑ downhill speed	Steep downhill racing (Defer et al., 2019)	May ↑ calf loading; not ideal for Achilles-sensitive runners
High Stack Height	↑ Cushioning, ↓ vibration, ↓ leg muscle demand	Long flat stages, smooth trails	Reduced lateral stability, slower foot reaction
Increased LBS (e.g., carbon plate)	↓ energy cost, maintains ankle propulsion longer	Fast runners, flat/mixed terrain (Rodrigo-Carranza et al., 2024)	May not benefit slower paces or untrained runners
Lighter Shoe Mass	↓ oxygen cost, ↓ swing phase effort	All trail types if durability and grip remain	Trade-off with protection and cushioning
AFT after habituation	↑ biomechanical efficiency, ↓ energy cost	Runners trained in curved-plate shoes (Matties et al., 2024)	May require 6–8 weeks of adaptation for benefit

### 5.1 Footwear selection is context-dependent

Research shows apparent inter-individual differences in how runners respond to footwear interventions. For example, Flores et al. (2019) identified distinct muscle activation strategies in runners who benefited from high-energy-return or LBS shoes, such as proximal (vastus medialis-dominant) versus distal (gastrocnemius-dominant) coordination. Similarly, Rodrigo-Carranza et al. (2023) reported that national-level runners showed greater improvements from high-stiffness footwear compared to sub-elite athletes, suggesting that training status, speed, and familiarity with a shoe design play a significant role in its effectiveness.

Therefore, recommendations should move beyond generalized labels like “minimalist” or “maximalist,” and instead emphasize functional fit: How does a shoe interact with a given runner’s technique under fatigue? How does it perform on the terrain profile of the target race? These questions should guide both research and practice.

### 5.2 Training for adaptability and resilience

Equally important is the runner’s capacity to adapt their biomechanics as fatigue sets in or as terrain changes. Studies by Kasmer et al. (2014) and Giandolini et al. (2016b) suggest that performance is supported not by rigid adherence to a particular strike pattern or cadence, but by the ability to modulate movement dynamically in response to external demands. Training should include components that develop proprioception, muscular robustness across joint ranges, and exposure to varied terrain, especially in the footwear intended for race day.

### 5.3 Wear progression and shoe lifecycle

Several studies have demonstrated that shoes degrade significantly throughout a single ultramarathon, particularly in terms of cushioning and shock absorption (Lloria-Varella et al., 2022). This degradation can affect impact loading and leg control even without visible wear. Thus, ensuring pre-race familiarization with footwear and monitoring functional degradation over time is essential. In events exceeding 8–10 h, rotating footwear or choosing midsole technologies with proven resilience may offer protective advantages. While in-race shoe changes are occasionally feasible in stage races or supported ultramarathons, most athletes will find rotation more practical across training blocks. Using multiple pairs allows midsole materials to recover between sessions, extending functional lifespan and reducing cumulative degradation on race day.

### 5.4 Terrain-specific considerations

Downhill-specific performance benefits have been demonstrated for low-drop footwear, which promotes a more anterior strike and greater speed during descent (Defer et al., 2019). On the other hand, higher stack height shoes may enhance comfort and cushioning for long-duration flats or gradual climbs but may compromise stability in rocky or technical terrain. Inappropriate matching of stack height or dropping into technical terrain may elevate the risk of ankle sprains or falls. Therefore, course profile and terrain type should inform shoe selection, race-day pacing, and gait strategy. These findings suggest that there is no one-size-fits-all solution in trail and ultramarathon footwear. Instead, success depends on tailoring design elements to the event’s demands and the unique biomechanical and neuromuscular characteristics of the runner. In particular, low-drop footwear, while advantageous for downhill efficiency, places an additional load on the Achilles tendon and calf complex, which can increase the risk of overuse in fatigued states.

Taken together, these considerations can be framed as a simple decision logic: if the course is predominantly downhill, low-drop shoes may facilitate faster descent but require sufficient Achilles resilience; if long flat or gradual climbs dominate, higher stack shoes may improve comfort and shock absorption but compromise stability on rocky terrain; for highly technical courses, lighter and lower-stack options may improve proprioception and balance. In practice, runners and coaches should weigh these factors against individual biomechanics and fatigue responses when finalizing footwear choice.

Beyond general shoe properties, runners should also account for individual characteristics such as arch height, habitual strike pattern, and ankle stability. In ultramarathon contexts, footwear strategies can be terrain-specific—for example, reserving cushioned, higher-stack shoes for prolonged flat terrain or climbs, while switching to lower-drop, more agile models for technical descents. Monitoring early signs of fatigue or gait changes during competition can further guide in-race adjustments, helping athletes strike a balance between efficiency and injury prevention.

### 5.5 Methodological considerations

The evidence base reveals clear methodological contrasts. Laboratory studies typically involve small samples of recreational or trained runners, ensuring internal validity but limiting ecological generalizability. Field-based ultramarathon studies, by contrast, often rely on observational designs and small elite samples, providing unique external validity but reduced methodological control. Broader determinants of running economy, including biomechanical, neuromuscular, and training-related factors, should also be considered when interpreting footwear effects (Saunders et al., 2004). Footwear-related findings also need to be interpreted within broader physiological frameworks of endurance performance, including oxygen transport, muscle metabolism, and neuromuscular fatigue (Joyner and Coyle, 2008).

Notably, Kasmer et al. (2014) observed a persistent rearfoot strike pattern in ultramarathoners, whereas Mo et al. (2021) reported no systematic differences in strike patterns across various trail conditions. These inconsistencies underscore the importance of integrating both laboratory precision and field realism in future work. Bridging these approaches represents one of the central challenges for advancing research in ultramarathon footwear biomechanics.

## 6 Conclusion

This review highlights that footwear has a substantial influence on biomechanics, running economy, and fatigue adaptation in trail and ultramarathon running. However, the evidence does not support a single universally optimal shoe. Instead, several central insights emerge:

• **Footwear effects are dynamic**–their benefits depend on terrain, race duration, and individual biomechanics rather than static design categories.
• **Fatigue affects both the runner and the shoe**–neuromuscular fatigue and midsole degradation interact, altering gait mechanics and load distribution over time.
• **Adaptability matters more than fixed technique**–successful ultramarathon runners rely on flexible strategies, adjusting strike pattern, cadence, and stability demands to match evolving conditions.

These principles suggest that future footwear research and athlete preparation should prioritize adaptability and context-specific strategies over universal prescriptions.

## 7 Limitations and future directions

While this review provides an integrative synthesis of biomechanical and performance-related effects of footwear in trail and ultramarathon running, several limitations must be acknowledged. This synthesis is based exclusively on 21 rigorously selected studies, which, although carefully chosen, still exhibit heterogeneity in study design, population characteristics, terrain conditions, and outcome measures. Methodological variability—such as treadmill versus field testing, short-term laboratory protocols versus in-race assessments, and differing approaches to fatigue measurement—limits the direct comparability of results across studies. In addition, many studies involved predominantly male, sub-elite or elite athletes, which may reduce the generalizability of findings to recreational or female runners. The majority of included studies were supportive laboratory trials, which limits ecological validity for ultramarathon conditions. Conclusions in this review, therefore, draw primarily on direct evidence, while mechanistic insights from supportive studies are interpreted cautiously.

Notably, one included study (Matties et al., 2024) was available only as a preprint at the time of analysis. Although it met all inclusion criteria and underwent full-text quality assessment, the absence of a formal peer review may limit confidence in its findings. To ensure transparency, this was indicated in the selection criteria.

Furthermore, most studies evaluated individual footwear characteristics in isolation, rather than exploring the interaction between multiple features (e.g., how midsole cushioning interacts with bending stiffness or shoe mass). Only a few studies have addressed midsole fatigue or long-distance material degradation, factors that are likely to affect real-world performance. Finally, due to methodological diversity, a quantitative meta-analysis was not conducted. While narrative synthesis allows contextual interpretation, it does not allow statistical pooling or effect size quantification.

## 8 Future directions

To address these gaps, future research should prioritize:

• Multifactorial study designs examining the combined influence of cushioning, LBS, drop, and shoe degradation across different terrains and race durations.
• Longitudinal field studies that track biomechanical and metabolic changes throughout full-length trail or ultramarathon events.
• Greater inclusion of female, master, and recreational athletes to improve the applicability of findings across the broader endurance community.
• Investigation into adaptive gait strategies and real-time biomechanical variability under fatigue, particularly concerning injury prevention and terrain transitions.
• Development of standardized protocols for measuring fatigue-related changes in both the runner and the shoe over time.
