# Supplementary material for: Footwear technology and biomechanical adaptations in ultramarathon running: a PRISMA-Guided narrative review integrating direct and laboratory evidence
Source: Front Bioeng Biotechnol. 2025 Sep 25;13:1642555. doi: 10.3389/fbioe.2025.1642555 (PMC12507829; doi:10.3389/fbioe.2025.1642555)
Supplement: Supplementary file 1 [file Table1.docx]

**Supplementary Table S1.** Methodological quality assessment of the included studies using the Modified Newcastle-Ottawa Scale (NOS).

| **Study (Author, Year)** | **Evidence Category** | **Selection (0–4)** | **Comparability**  **(0–2)** | **Outcome/**  **Exposure (0–3)** | **Total Score (0–9)** | **Quality Classification** |
| --- | --- | --- | --- | --- | --- | --- |
| Arshad et al. (2022) | Direct | 3 | 2 | 3 | 8 | High |
| Bartolomé et al. (2021) | Supportive | 3 | 2 | 2 | 7 | Moderate |
| Bastone et al. (2020) | Supportive | 2 | 2 | 3 | 7 | Moderate |
| Fuller et al. (2015) | Supportive | 3 | 1 | 3 | 7 | Moderate |
| Gerodimos et al. (2021) | Supportive | 3 | 2 | 2 | 7 | Moderate |
| Hoogkamer et al. (2018) | Supportive | 4 | 2 | 3 | 9 | High |
| Kasmer et al. (2014) | Direct | 3 | 1 | 3 | 7 | Moderate |
| Matties et al. (2024, preprint) | Supportive | 2 | 1 | 2 | 5 | Low |
| Millet (2011) | Supportive | 3 | 2 | 3 | 8 | High |
| Mo et al. (2021) | Direct | 3 | 2 | 3 | 8 | High |
| Nigg & Enders (2013) | Supportive | 3 | 2 | 2 | 7 | Moderate |
| Perl et al. (2012) | Supportive | 3 | 1 | 3 | 7 | Moderate |
| Radder et al. (2019) | Direct | 4 | 2 | 3 | 9 | High |
| Rodrigo-Carranza et al. (2023) | Direct | 3 | 2 | 2 | 7 | Moderate |
| Rodrigo-Carranza et al. (2024) | Direct | 3 | 2 | 2 | 7 | Moderate |
| Saunders et al. (2004) | Supportive | 3 | 2 | 3 | 8 | High |
| Shorten (2024) | Supportive | 2 | 1 | 2 | 5 | Low |
| Tanaka et al. (2020) | Direct | 3 | 2 | 3 | 8 | High |
| Trama et al. (2023) | Direct | 3 | 2 | 3 | 8 | High |
| Joyner & Coyle (2008) | Supportive | 3 | 2 | 3 | 8 | High |
| Zulham et al. (2024) | Direct | 3 | 1 | 3 | 7 | Moderate |

Each of the 21 included studies was a independently assessed by two reviewers based on a full-text analysis using a modified version of the NOS, adapted for biomechanical and performance-oriented research. The assessment consisted of three domains: Selection (maximum 4 points), Comparability (maximum 2 points), and Outcome/Exposure Assessment (maximum 3 points), resulting in a total score ranging from 0 to 9. Based on total scores, studies were categorized as high quality (8–9 points), moderate quality (6–7 points), or low quality (≤5 points). Studies are listed in alphabetical order by first author.
